# Gibberellic Acid: A Key Phytohormone for Spikelet Fertility in Rice Grain Production

**DOI:** 10.3390/ijms17050794

**Published:** 2016-05-23

**Authors:** Choon-Tak Kwon, Nam-Chon Paek

**Affiliations:** 1 Department of Plant Science, Plant Genomics and Breeding Institute, and Research Institute of Agriculture and Life Sciences, Seoul National University, Seoul 151-921, Korea; lifemir7@snu.ac.kr; 2 Crop Biotechnology Institute, GreenBio Science and Technology, Seoul National University, Pyeongchang 232-916, Korea

**Keywords:** gibberellin signaling, rice, stamen/anther development, spikelet fertility, grain productivity

## Abstract

The phytohormone gibberellic acid (GA) has essential signaling functions in multiple processes during plant development. In the “Green Revolution”, breeders developed high-yield rice cultivars that exhibited both semi-dwarfism and altered GA responses, thus improving grain production. Most studies of GA have concentrated on germination and cell elongation, but GA also has a pivotal role in floral organ development, particularly in stamen/anther formation. In rice, GA signaling plays an important role in spikelet fertility; however, the molecular genetic and biochemical mechanisms of GA in male fertility remain largely unknown. Here, we review recent progress in understanding the network of GA signaling and its connection with spikelet fertility, which is tightly associated with grain productivity in cereal crops.

## 1. Introduction

The phytohormone gibberellic acid (GA; also referred to as gibberellin) regulates almost all processes of plant development and growth, including seed development and germination, stem and root growth, cell division, and flowering time. When first discovered, GA was named after a fungus *Gibberella fujikuroi* that causes uncontrolled cell elongation and various disease symptoms in infected plants [1,2,3]. Numerous GA compounds have been identified in plant tissues, but *in vivo*, most GA occurs as biologically inactive forms [4]. In reproductive development, GA has essential functions in stamen/anther formation [5,6,7,8], pollen formation, and pollen tube development [9,10,11].

Screening for GA-insensitive and GA-deficient mutants, which generally have dwarf or slender phenotypes, has identified many genes that function in GA-responsive signaling in *Arabidopsis* and rice [12] and analysis of these genes has shown conservation of GA signaling pathways [13,14]. In particular, several factors involved in GA signaling have high protein sequence similarity and play similar roles in stamen development in *Arabidopsis* and rice, which indicates the existence of a conserved pathway in stamen/anther development in angiosperms [15,16,17,18]. The identification and functional analyses of GA-related genes has improved our understanding of GA signal transduction [19]. In this review, we describe recent progress in revealing the molecular mechanisms of GA signaling in stamen/anther development and spikelet fertility in rice.

## 2. Gibberellic Acid (GA) Signal Transduction in Rice Development

**Figure 1:**
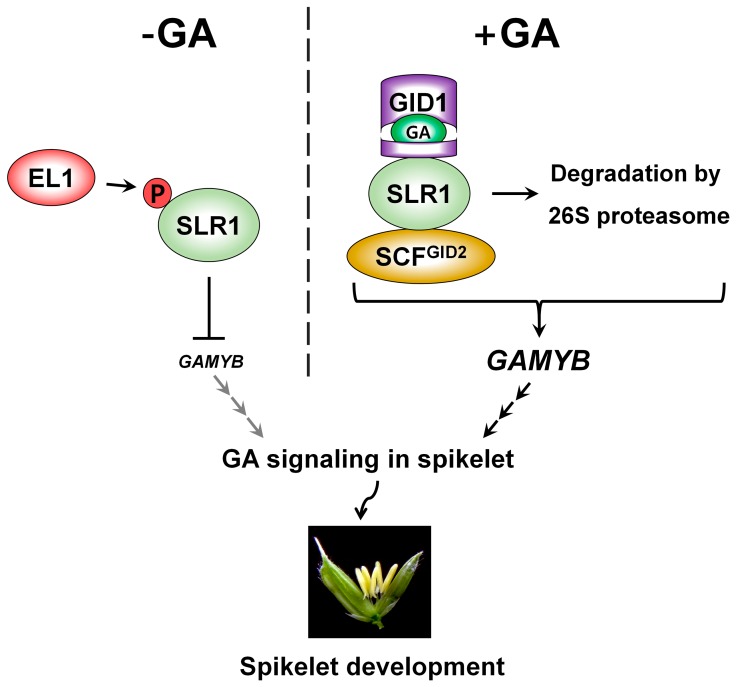
Gibberellic acid (GA) signaling in spikelet development. Under GA-deficient conditions, the casein kinase I (EL1) phosphorylates SLENDER RICE1 (SLR1) to increase its activity and stability; then, SLR1 represses the expression of *GAMYB*. Under GA-sufficient conditions, GA is perceived by GID1 and the resulting conformational change of GID1 allows it to interact with and degrade SLR1 through a SCF^GID2^-mediated proteasome pathway. In the absence of SLR1 activity, *GAMYB* transcription becomes de-repressed; then, GAMYB protein positively regulates the transcription of several downstream genes for formation of the Ubisch body and exine, as well as for cell death in the tapetum during spikelet development.

In GA signaling, a central DELLA protein inhibits GA-associated development, and GA induces the protease-mediated degradation of DELLA [3,20]. In rice, (see schematic in Figure 1) the DELLA protein SLENDER RICE1 (SLR1) negatively regulates downstream genes in the GA signaling pathway, including *GAMYB*, which encodes a transcription factor that positively regulates GA-responsive genes (Table 1) [12,21,22]. In the current model of GA signal transduction, the biologically active GA_4_ directly binds to GA-INSENSITIVE DWARF1 (GID1), a soluble GA receptor (Table 1) [23], and the GA-GID1 complex interacts with [24] and degrades SLR1 through the 26S proteasome pathway, mediated by the Skp1, Cullin, F-box complex with the F-box protein subunit GA-INSENSITIVE DWARF2 (SCF^GID2^ complex) (Table 1) [25,26]. SLR1 functions as the central negative regulator in GA signaling and exists in phosphorylated and non-phosphorylated forms *in vivo* [25,26,27]. It was initially proposed that degradation of SLR1 by the SCF^GID2^ complex depends on its phosphorylation [25,26]. However, interaction of non-phosphorylated SLR1 and GID2 also occurs *in vivo*, suggesting that the phosphorylation status is not a prerequisite for degradation of SLR1 by the SCF^GID2^ complex [27]. Despite recent progress in studies of the post-translational modification of SLR1, the factors involved in the post-translational regulation of SLR1 stability remain elusive. *Rice SPINDLY* (*OsSPY*) encoding an O-linked *N*-acetylglucosamine transferase does not control SLR1 stability but probably functions to repress SLR1 function in GA signaling (Table 1) [28]. Recent work reported that *Early flowering1* (*EL1*) encodes a casein kinase I that functions in the negative regulation of GA signaling by phosphorylating and activating SLR1 (Table 1) [29,30]. In addition, the phosphorylation on N-terminus of SLR1 inhibits GID1-mediated degradation, and phosphorylation on C-terminus maintains SLR1 to be an active form [29]. Although the phosphorylation of SLR1 by EL1 contributes to maintaining the stability and activity of SLR1 [29], the biological significance of SLR1 phosphorylation remains to be determined.

The degradation of SLR1, caused by GA perception, de-represses the transcription of downstream genes in the GA signaling pathway (Figure 1). GAMYB is the main transcription factor in GA signaling and activates α-amylase gene expression in the cereal aleurone layers during germination (Table 1) [31,32,33,34,35]. GAMYB also functions in stamen/anther and pollen development in angiosperms (Figure 1) [10,12,35,36,37]. The *gamyb* knockout mutants in rice showed severe defects in spikelet development, particularly in the formation of anthers and pollen [35]. GAMYB activates the expression of downstream genes that regulate exine and Ubisch body formation in pollen by directly binding to their promoters [12]. Thus, GA signaling plays a central role in floral organ formation.

## 3. Roles of GA Signaling in Male Organ Development in Rice

Development of stamens and anthers requires GA signaling, as malfunction of GA signaling results in male sterility. Most GA-insensitive or -deficient mutants in rice, petunia, maize, tomato, barley and *Arabidopsis* exhibit defective stamen/anther structure, which often produces sterile flowers [5,7,10,12,21,35,38,39,40,41,42]. Particularly in rice, the genes involved in GA signaling have key functions in anther and pollen formation [10,12,35]. For example, the rice GA-insensitive *gid1-4* and *gid2-5* mutants show deformation of pollen mother cell (PMC) tetrads during anther development [12]. Although the exact reasons for the PMC disruption remain unknown, GID1- and GID2-dependent GA signaling is necessary to form the structure of anthers. In addition, the rice GA-deficient mutant *ent-copalyl diphosphate synthase1-1* (*oscps1-1*) produces defective stamens with whitened and shrunken anthers and abnormal tapetum [12,43]. DELLA proteins, the main repressors of GA signaling, also affect other hormone signaling, and thus it is possible that additional mechanisms associated with DELLAs affect stamen development [18,44,45,46]. In most GA mutants, one of the structural deformities of anthers is hypertrophy of tapetal cells, indicating that GA signaling is involved in tapetum degeneration [12]. For instance, both *oscps1-1* and *gid1-4* mutants in rice show a morphological abnormality of the tapetum, although it is uncertain how GA-deficiency induces the phenotype [12,18].

The formation of male reproductive organ in plants is influenced by numerous internal factors, including microspore maturation, meiosis, differentiation of sporogenous cells, and stamen specification [47,48,49,50]. Transcriptome analyses showed that any transcripts are expressed in each developmental stage of anther and male gametophyte development in rice [50], and some of these transcripts indicate that GA signal transduction occurs in developing microspores and tapetum cells within the anther [51,52]. Microarrays and comprehensive network analyses of the genes expressed in anther have identified some target genes for GA signaling in rice, such as *CYP703A3* and *KAR* encoding a cytochrome P450 hydroxylase and β-ketoacyl reductase, respectively [12,53].

As mentioned above, the GA signaling component GAMYB is negatively regulated by SLR1 and acts in anther development as a key transcription factor by directly binding to the promoters of several GA-responsive genes that function in the formation of exine and the Ubisch body as well as the degeneration of tapetal cells [12,35]. The *gamyb-1* mutants have defective anthers [35] and the transcription of *CYP703A3* and *KAR*, which are responsible for pollen formation, is dramatically suppressed in *gamyb-2* mutants, resulting in defective anther development [12]. Microarray data showed that GAMYB also positively controls the expression of a key gene for tapetum degradation, *TAPETUM DEGENERATION RETARDATION* (*TDR*), during anther development [12,54]. Other genes are also necessary for programmed cell death of tapetal cells, such as *OsC6* and *OsCP1,* encoding a lipid transfer protein and a cysteine protease, respectively; TDR directly controls expression of these genes [54,55]. Additionally, GAMYB appears to be a positive regulator of *OsC6* by directly interacting with its promoter [12]. Thus, these results suggest that precise regulation of *GAMYB* expression is necessary for the normal development of male reproductive organs and thus for spikelet fertility in rice (Figure 1).

## 4. The Role of GA in Rice Spikelet Fertility

Spikelet fertility is a critical yield-determining trait that is influenced by genetic background and environmental factors including rain, wind, and temperature. Stamen/anther development and pollen viability are essential for fertility of spikelets [56]. Numerous genetic studies have revealed the functions of many genes controlling formation of pollen and anthers. In particular, rice varieties with unregulated GA-induced signaling showed a sterile phenotype due to defects of anther and pollen formation. For instance, loss-of-function *slr1-1* and gain-of-function *Slr1-d3* mutants displayed sterile and semi-fertile phenotypes, respectively [10,21]. Although *Slr1-d3* mutants produced normal floral organs with morphologically normal pistils and stamens, their pollen had very low viability, leading to a semi-fertile phenotype [10]. In addition, *gamyb* null mutants displayed fewer spikelets per panicle, increased male sterility, and decreased expression of α-amylase genes in response to application of GA [35].

Although recent studies of GA signaling have focused on the consequences of GA deficiency in stamen development, defects in GA signaling also negatively affect silique fertility in *Arabidopsis* [57]. Several GA-deficient mutants in plants develop defective anthers, leading to male sterility [5,7]. Overexpression of GA-related genes also often leads to male sterility and failure to set seed; for example, transgenic barley overexpressing *HvGAMYB* show increased male sterility, resulting in loss of grain production [58]. Overexpression of a pea *GA 2-oxidase 2* gene induced seed abortion in *Arabidopsis* [9]. In *Arabidopsis*, a loss of DELLA activity in the Columbia ecotype caused male sterility and defective fertility due to post-meiotic problems in pollen formation but caused no defect in the Landsberg *erecta* (L*er*) ecotype [57]. In monocot plants such as rice and barley, loss of DELLA (SLR1/SLN1) function leads to a sterile phenotype [10,21,38]. The plants overexpressing an antisense construct that targets *OsSPY*, which encodes a factor that post-translationally modifies SLR1, developed few fertile spikelets [28]. Recently, we found that a defect of *EL1*, a casein kinase I phosphorylating SLR1, negatively affects spikelet fertility in rice [11].

In plants, several mutants exhibiting male sterility are sensitive to temperature and photoperiod, suggesting that the response to environmental conditions also closely associates with development of reproductive organs [59]. For example, compared with other stages, the early stage of anther development is more sensitive to low and/or high temperature in cereal crop plants including rice [60,61]. In particular, reduction of the levels of active GA forms in response to low temperature causes abnormal pollen development, leading to decreased spikelet fertility [61]. Thus, these observations strongly suggest that a significant connection exists between GA synthesis and environmental stresses during reproductive organ development.

## 5. Conclusions

Rice (*Oryza sativa* L.) is a staple food for more than a half of the world population, mainly in Asia. To increase rice grain production, yield-related components such as panicle number per plant, spikelet number per panicle, spikelet fertility, and grain weight need to be improved. In addition, it will be crucial to maintain the balance of agronomic traits; for instance, excessive tillering often gives rise to a decrease in grain production, because tillers can contend with the main culm for resources and negatively affect seed filling rate [56]. Likewise, plant hormones and the genes that are involved in hormone biosynthesis or signaling affect anther dehiscence and pollen maturation, which are closely related to grain productivity. Auxin and jasmonic acid are also crucial for spikelet fertility and regulate anther and pollen development similar to GA [62,63,64,65].

There are many open questions about GA and spikelet development in rice. First, it is as yet unknown what the SLR1-interacting factor(s) and direct target(s) of SLR1 are in the stamen during spikelet development. DELLAs directly interact with several proteins, such as PIF, MYC2, JAZ, and EIN3 in other hormone pathways in *Arabidopsis* [66,67,68,69,70]. However, there are no reports of the functions of these factors during stamen development in rice and *Arabidopsis*. Thus, it is possible that stamen growth is related to the crosstalk between GA and other hormone signaling pathways. Furthermore, yet-undiscovered gene(s) also likely affect stamen development. In the GA signaling cascade, GAMYB is a central regulator of anther development. Several direct target genes of GAMYB in anther development have been established [12], but the direct transcriptional regulator of *GAMYB* downstream of *SLR1* remains to be identified. In *Arabidopsis*, DELLA activity in the L*er* ecotype is not essential to maintain seed fertility; however, the mutation of *DELLA* genes in Col-0 ecotype induces male sterility, due to post-meiotic defects in pollen formation. [57,71,72]. Recent work in rice found that a deficiency of SLR1 activity causes different spikelet fertility phenotypes in two different cultivars, “Koshihikari” and “H143” [11]; H143 exhibits partial sterility of spikelets while Koshihikari has normal fertility, even though these two cultivars have the same mutant allele of *EL1*, and thus have defects in EL1, the kinase that phosphorylates SLR1 to increase its activity and stability [11,29]. These findings suggest that different pathways downstream of *SLR1*, or completely unknown regulatory mechanisms, may govern spikelet development. Therefore, further studies to find as-yet-undetermined mechanisms are necessary to expand our knowledge of GA signaling in spikelet fertility.

Furthermore, analyses of the relationship between plant hormones and grain productivity are necessary to pioneer crop science in the future. In addition, world climate change will likely have a major effect on the grain productivity of many crops. We currently know the relationship between environmental cues and GA in reproductive organ formation. However, the molecular analysis of those relationships is still at an early stage. Therefore, a deeper understanding will provide more insights to help in the improvement of crop productivity.

## Figures and Tables

**Table 1:** Genes involved in gibberellic acid (GA) signaling in rice.

Gene	MSU ID ^a^	RAP ID ^b^	Function	Conserved Domain	Polypeptide	Reference
*SLR1*	Os03g49990	Os03g0707600	Transcription regulation	DELLA, GRAS	625 aa	[21]
*GID1*	Os05g33730	Os05g0407500	GA receptor	Hormone sensitive lipase (HSL)	354 aa	[23]
*GID2*	Os02g36974	Os02g0580300	DELLA degradation	F-box	212 aa	[25]
*EL1*	Os03g57940	Os03g0793500	Phosphorylation	Ser/Thr kinase	707 aa	[29]
*GAMYB*	Os01g59660	Os01g0812000	Transcription factor	Myb	553 aa	[32]
*OsSPY*	Os08g44510	Os08g0559300	O-GlcNAcylation	O-GlcNAc transferase	927aa	[28]

^a^ Data from Michigan State University; ^b^ Data from Rice Genome Annotation Project.

## Abbreviations

GA	Gibberellic acid
SLR1	Slender rice1
GID1	GA INSENSITIVE DWARF1
GID2	GA INSENSITIVE DWARF2
SCF	Skp, Cullin, F-box
OsSPY	Rice SPINDLY
EL1	Early flowering1
PMC	Pollen mother cell
OsCPS	Rice ent-copalyl diphosphate synthase
CYP703	Cytochrome P450 hydroxylase 703
KAR	β-Ketoacyl reductase
TDR	TAPETUM DEGENERATION RETARDATION
OsCP	Rice cysteine protease
Col-0	Columbia
L*er*	Landsberg *erecta*
PIF	Phytochrome-interacting factor
JAZ	Jasmonate ZIM-domain
EIN3	Ethylene insensitive3
